# Escalas para valoración del dolor neonatal: Una revisión integrativa*

**DOI:** 10.15649/cuidarte.2760

**Published:** 2023-09-07

**Authors:** Gustavo Edgardo Jiménez Hernández, Javier Alonso Bula Romero, Álvaro Antonio Sánchez Caraballo, Martín Elías Peña Zuluaga

**Affiliations:** 1 Universidad de Córdoba, Monteria, Colombia. Email: gustavojimenezh@correo.unicordoba.edu.co Universidad de Córdoba Universidad de Córdoba Monteria Colombia gustavojimenezh@correo.unicordoba.edu.co; 2 Universidad de Córdoba, Monteria, Colombia. Email: javierbula@correo.unicordoba.edu.co Universidad de Córdoba Universidad de Córdoba Monteria Colombia javierbula@correo.unicordoba.edu.co; 3 Universidad de Córdoba, Monteria, Colombia. Email: aasanchez@correo.unicordoba.edu.co Universidad de Córdoba Universidad de Córdoba Monteria Colombia aasanchez@correo.unicordoba.edu.co; 4 Universidad de Córdoba, Monteria, Colombia. Email: bymach1@gmail.com Universidad de Córdoba Universidad de Córdoba Monteria Colombia bymach1@gmail.com

**Keywords:** Escalas, Evaluación del Dolor, Recién Nacido, Scales, Pain Measurement, Newborn, Escalas, Medigáo da Dor, Recém-Nascido

## Abstract

**Introducción:**

Debido a que los niños en la etapa de desarrollo preverbal no pueden expresar sus sentimientos, las escalas para valorar el dolor en neonatos son una buena herramienta para uso en la práctica clínica por el profesional de enfermería.

**Objetivo:**

Describir las escalas válidas y confiables que se utilizan en la práctica de enfermería para evaluar el dolor en neonatos.

**Materiales y métodos:**

Se realizó una revisión integrativa de literatura durante los años 2019 a 2020 de artículos publicados desde el año 1990. Las bases de datos consultadas fueron: PubMed, Lilacs, Proquest, Science Direct, Embase, BVS, Scopus y el metabuscador Google académico. Se analizaron 22 artículos que cumplieron con los criterios de inclusión para su respectivo análisis.

**Resultados:**

Se encontraron instrumentos unidimensionales y multidimensionales para la evaluación del dolor agudo y prolongado en recién nacidos prematuros y a término.

**Discusión:**

Esta revisión integrativa proporciona a los profesionales de salud, y en especial al profesional de enfermería bases conceptuales para la implementación de herramientas de evaluación clínica del dolor, según la edad gestacional, la duración del dolor y el tipo de indicador.

**Conclusiones:**

De acuerdo al conocimiento y características de las escalas, no se puede establecer claramente la más adecuada para uso general o patrón de oro, la selección dependerá de diferentes criterios, como tipo de estímulo, la edad gestacional, y del contexto en el que se encuentra el recién nacido.

## Abstract (en)

**Introduction:**

Since infants are not able to express their feelings during the preverbal stage of development, neonatal pain rating scales are good tools for nurses to use in clinical practice.

**Objective:**

To describe valid and reliable scales used in nursing practice to assess neonatal pain.

**Materials and Methods:**

An integrative review of articles published since 1990 was conducted from 2019 to 2020. The databases consulted were PubMed, Lilacs, ProQuest, ScienceDirect, Embase, VHL, Scopus, and Google Scholar metasearch engine. Twenty-two articles that met the inclusion criteria were analyzed.

**Results:**

Unidimensional and multidimensional instruments were found for assessing acute and prolonged pain in preterm and term neonates.

**Discussion:**

This integrative review provides healthcare professionals, especially nurses, with a conceptual basis for implementing clinical pain assessment tools according to gestational age, duration of pain, and type of indicator.

**Conclusions:**

According to the knowledge and characteristics of the scales, it is not possible to clearly establish the most appropriate one for general use or a gold standard; the selection will depend on different criteria, such as the type of stimulus, gestational age, and the newborn's context.

## Resumo

**Introdujo:**

Como as crianzas na fase pré-verbal do desenvolvimento nao conseguem expressar seus sentimentos, as escalas de avaliagáo da dor em neonatos sao uma boa ferramenta para uso na prática clínica pelos profissionais de enfermagem.

**Objetivo:**

Descrever as escalas válidas e confiáveis utilizadas na prática de enfermagem para avaliar a dor em neonatos.

**Materiais e Métodos:**

Foi realizada uma revisao integrativa da literatura durante os anos de 2019 a 2020 de artigos publicados desde 1990. As bases de dados consultadas foram: PubMed, Lilacs, Proquest, Science Direct, Embase , BVS, Scopus e o metabuscador académico Google. Foram analisados 22 artigos que atenderam aos critérios de inclusao para sua respectiva análise.

**Resultados:**

Foram encontrados instrumentos unidimensionais e multidimensionais para avaliagáo da dor aguda e prolongada em recém-nascidos pré-termo e a termo.

**Discussao:**

Esta revisáo integrativa fornece aos profissionais de saúde, e especialmente aos profissionais de enfermagem, bases conceituais para a implementagáo de instrumentos de avaliagáo clínica da dor, de acordo com a idade gestacional, duragáo da dor e tipo de indicador.

**Condusdes:**

De acordo com o conhecimento e as características das escalas, náo é possível estabelecer claramente a mais adequada para uso geral ou padráo-ouro, a selegáo dependerá de diferentes critérios, como tipo de estímulo, idade gestacional e contexto em que é usado. o recém-nascido é encontrado.

## Introducción

En los recién nacidos la capacidad de comunicación verbal es inexistente. Por tanto, no es posible conocer el grado de dolor que presentan ante un procedimiento diagnóstico o terapéutico durante su atención^1^^,^^2^; situación que se convierte en un reto para el profesional de enfermería al momento de brindar cuidado. Es ahí, donde las escalas de medición del dolor se vuelven herramientas útiles para aplicar y estimar el grado de dolor que muestren los recién nacidos, teniendo en cuenta parámetros de tipo fisiológico, bioquímico y comportamental.

Solo hasta la década de los años 60 se empezó a caracterizar el dolor neonatal. A partir de este período surge evidencia científica, que describe los cambios fisiológicos y bioquímicos que presentan los neonatos ante la exposición a estímulos dolorosos^3^. Por lo tanto, los avances en áreas de pediatría y neonatología en los estudios para valorar el dolor han sido cada vez mayor^4^^,^^5^; incluso en los últimos 40 años se han desarrollo un número considerable de escalas, que por su sensibilidad han permitido valorar el dolor de acuerdo a las características y condiciones neonatales, permitiendo así un mejor abordaje del cuidado, producto de procedimientos clínicos de rutina o más complejos^6^^,^^7^.

Para el profesional de enfermería del área neonatal, evaluar el dolor constituye un importante reto en la práctica clínica^2^^,^^8^^,^^9^. La evidencia científica ha demostrado que este profesional reconoce la presencia del dolor en los neonatos. Sin embargo, la implementación de escalas de medición para valorarlo no es frecuente, dado que, el dolor en neonatos, es valorado desde la práctica empírica^2^^,^^10^^,^^11^.

Los neonatos en el contexto hospitalario experimentan de manera rutinaria experiencias de dolor, entre otras razones por la manipulación a la que éste es expuesto por procedimientos terapéuticos y diagnósticos^12^; dentro de los que se destacan, punción del talón, toma de muestras para laboratorio clínico y venopunción, además del dolor que puede ser causado por la sintomatología de base; de ahí que, valorar el dolor en los neonatos permite establecer parámetros del estado fisiológico, orientando la práctica de enfermería y el desarrollo de competencias instrumentales que contribuyan a generar el mínimo dolor posible^13^.

Desde el punto de vista teórico, esta revisión de la literatura profundiza en el conocimiento existente de las escalas utilizadas en el ámbito clínico para valorar el dolor en los neonatos. Además, se ratifican las ventajas y limitaciones que puedan generar por sus características y aplicaciones. Lo cual puede permitir alternativas en la práctica de enfermería para la selección de la escala de acuerdo a su validez y adaptación cultural. También, la revisión posibilita conocer y relacionarse con diversas escalas como indicadores clínicos válidos y confiables. Convirtiéndose, en herramientas indispensables para la toma de decisiones en la práctica del cuidado neonatal.

## Materiales y Métodos

Se realizó una revisión integrativa de la literatura de tipo descriptivo, de acuerdo a los parámetros establecidos por Whittemore & Knaf^14^, y los criterios de la declaración PRISMA. Ésta se desarrolló en seis etapas como lo plantea Mendes et al. 2008^15^.

### Primera etapa

El problema planteado en esta revisión surgió a partir de la siguiente pregunta de investigación: ¿Qué evidencia teórica y empírica existe acerca del uso de escalas para valorar el dolor neonatal en el ámbito clínico?

### Segunda etapa

Se establecieron los criterios de inclusión y exclusión de esta revisión. La búsqueda y selección de los artículos científicos se inició en las bases de datos elegidas para tal fin. Los criterios de selección de las fuentes primarias fueron: artículo de revistas indexadas, en idioma inglés, español y portugués, en el periodo comprendido entre 1990 y 2020. Las bases de datos consultadas fueron: PubMed, Lilacs, Proquest, Science Direct, Embase, Bvs, Scopus y el metabuscador Google académico. Inicialmente, se realizó búsqueda exploratoria en los metabuscadores utilizando los siguientes descriptores: Scales, pain assessment, newborn; y su respectiva traducción al español y al portugués. Se consideraron los estudios en población de neonatos; no se aplicó un rango de edad estricto. Las intervenciones de interés fueron las que aplicaron una escala de valoración del dolor en los neonatos. En cuanto a los criterios de exclusión se tuvieron en cuenta: publicaciones de trabajos duplicados, artículos en los que no se aplicó una escala de valoración del dolor, literatura gris, artículos no publicados en revistas indexadas y sin disponibilidad de su resumen.

### Tercera etapa

Se realizó la lectura en texto completo de los artículos de investigación seleccionados, durante esta etapa se establecieron dos componentes: primero, descripción de las dimensiones y características psicométricas de las escalas utilizadas para valorar el dolor neonatal en el ámbito clínico y la identificación de estudios de validación o adaptación transcultural, al menos, en una población y/o ámbito clínico diferente al del estudio primario, y el segundo consistió en el análisis de las escalas desarrolladas por la disciplina de enfermería.

La pérdida de datos fue controlada por medio de la categorización de los artículos, para ello los investigadores diseñaron una matriz en Microsoft Excel con el fin de reunir la información. Por su parte, el sesgo de clasificación fue controlado manteniendo el rigor metodológico de las revisiones integrativas, siguiendo los pasos propuestos por Whittemore & Knaf^14^.

### Cuarta etapa

Se evaluaron los artículos seleccionados; los resultados de la evaluación fueron categorizados para dar respuesta a los objetivos planteados; aquí se realizó una lectura crítica de los artículos que documentaban las escalas utilizadas para valorar el dolor neonatal; procedimiento que fue crucial para la toma de decisiones, la integración y síntesis de la evidencia empírica documentada. La base de datos de artículos seleccionados fue almacenada en Mendeley Data^16^.

### Quinta etapa

Correspondió a la discusión, en ella se interpretaron los resultados a la luz de los hallazgos reportados y la evidencia empírica disponible; para la discusión se tuvo en cuenta otros estudios que documentaban el fenómeno, contrastando los resultados y definiendo si el número de escalas utilizadas para valorar el dolor neonatal, coincidía o no con otras revisiones de la literatura existente.

### Sexta etapa

Se establecieron las conclusiones e implicaciones de esta revisión para la práctica clínica en el ámbito de la enfermería neonatal, indicando ventajas y limitaciones existentes y proporcionando algunas sugerencias para futuras investigaciones.

**Gráfico 1:**
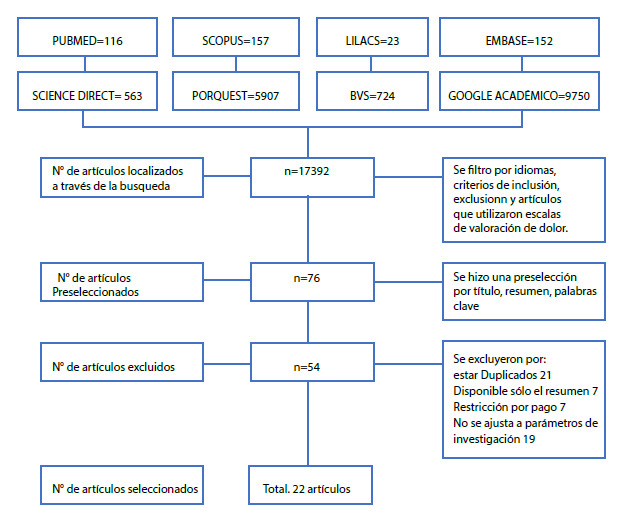
Declaración PRISMA

La búsqueda de los artículos se llevó a cabo por cuatro revisores de manera independiente; inicialmente se encontraron 17392 artículos, que luego de aplicar los filtros por idioma se preseleccionaron 76 para lectura completa; se excluyeron 54 artículos (21 artículos repetidos, 7 artículos que no estaban disponibles en texto completo, 7 artículos con restricción por pago, 18 artículos que no se ajustan al fenómeno de investigación, porque se trataban de estudios de correlación, donde se hacía uso de la escala, pero no se profundizaba sobre los aspectos psicométricos de la misma), finalmente, se seleccionaron 22 artículos para la revisión integrativa. Ver Gráfico 1 de la estrategia PRISMA.

**Gráfico 2:**
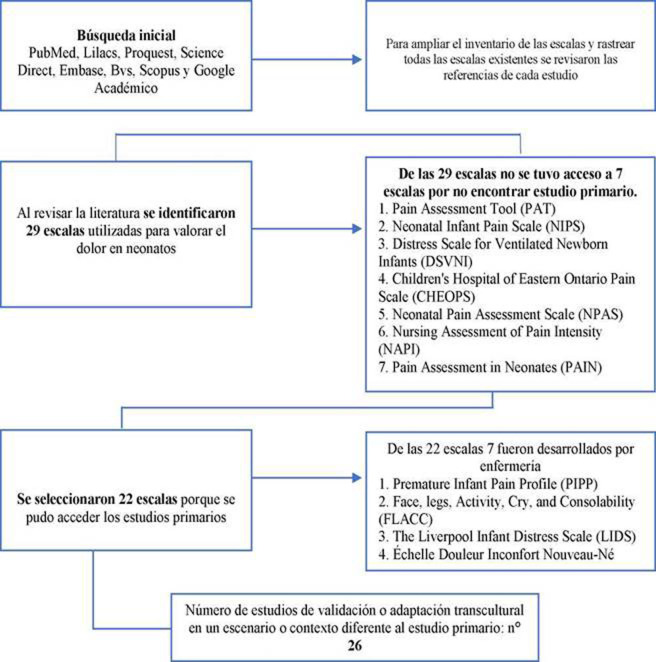
Flujograma de la toma de decisiones para la selección de las escalas utilizadas para valorar el dolor neonatal

Fuente: Análisis bibliométrico de las bases de datos consultadas.

## Resultados

El 49% de las escalas seleccionadas en esta revisión de la literatura fue desarrollada en América del Norte, en Oceanía (9%) y Asia con un 4% (Tabla 1). Los estudios primarios se publicaron en inglés. Además, hay referencias de estudios primarios en alemán^17^, francés^18^ y español^19^

Las 22 escalas seleccionadas para la valoración del dolor neonatal en el ámbito clínico (ver Gráfico 2), cuentan con estudios de validez y confiabilidad. En la mayoría de estas escalas se evaluó la consistencia interna a través del alfa de Cronbach y otros procedimientos psicométricos como armonía interjueces, metodología Delphi, metodología Q y pruebas de test-retest^20^

**Tabla 1:** Publicaciones incluidas de escalas utilizadas en el ámbito clínico para valorar el dolor neonatal

N°	Nombre de la escala	Autores	País de origen	Disciplina donde se creó la escala	Consistencia Interna	Clasificación
1	Neonatal Facial Coding system (NFCS)	Grunau & Craig	Canadá	Medicina (Pediatría)	0.88	Unidimensional
2	COMFORT	Ambuel et al	Estados unidos	Medicina (Pediatría)	0.84	Multidimensional
3	The Infant Body Coding System (IBCS)	Craig et al	Canadá	Psicología	0,83	Multidimensional
4	C-Crying; R-Requires increased oxygenadministration; I-Increased vital signs; E-Expression; S- Sleeplessness. (CRIES)	Krechel & Bildner	Estados unidos	Medicina (Anestesiología)	0.73	Multidimensional
5	Premature Infant Pain Profile (PIPP)	Stevens et al	Canadá	Enfermería (Pediatria)	0.95	Multidimensional
6	Douleur aigue chez le nouveau-né a terme et prématuré (DAN)	Carbajal et al	Francia	Medicina (Pediatria)	0.88	Unidimensional
7	Face, legs, Activity, Cry, and Consolability (FLACC)	Merkel et al	Estados unidos	Enfermería (Anestesiología)	0,71	Unidimensional
8	Children's and Infants Postoperative Pain Scale (CHIPPS)	Buettner & Finke	Alemania	Medicina (Anestesiología)	0.92	Unidimensional
9	The Liverpool Infant Distress Scale (LIDS)	Horgan et al	Inglaterra	Enfermería (Pediatria)	0,88	Unidimensional
10	Scale for use in Newborns (SUN)	Blauer et al	Estados unidos	Enfermería (Neonatología)	0,8	Multidimensional
11	Llanto, actitud, normorrespiración, tono postural y observación facial (LLANTO)	Herrera et al	España	Medicina (Anestesiología)	0.82	Unidimensional
12	Neonatal pain, agitation and sédation Scale (N-PASS)	Hummel et al	Estados unidos	Medicina (Perinatología)	0.82	Multidimensional
13	Échelle Douleur Inconfort Nouveau-Né (EDIN)	Debillon, et al	Francia	Enfermería (Pediatria)	0,92	Unidimensional
14	Nepean Neonatal Intensive Care Unit Pain Assessment Tool (NNICUPAT)	J Marceau	Australia	Medicina (Pediatria)	0,97	Multidimensional
15	(A) pitch of the first cry; (B) rhythmicity of the crying bout; (C) constancy of crying intensity (ABC)	Bellieni et al	Italia	Medicina (Pediatria)	0.77	Unidimensional
16	Behavioral Indicators of Infant Pain (BIIP)	Holsti & Grunau	Canadá	Medicina (Pediatria)	0.82	Unidimensional
17	Multidimensional Assessment of Pain Scale (MAPS)	Ramelet et al	Australia	Enfermería (Materno Infantil)	0,77	multidimensional
18	COMFORT B	Ista et al	Estados unidos	Medicina (Cirugía Pediátrica)	0.84	Unidimensional
19	Pain Assessment Scale for preterm infants (PASPI)	Liaw et al	Taiwán	Enfermería (Pediatria)	0.84	multidimensional
20	Astrid Lind gren Children's Hospital Pain Scale (ALPS NEO)	Lundqvist et al	Suecia	Medicina (Pediatria)	0.95	Unidimensional
21	Premature Infant Pain Profile revised (PIPP-R)	Stevens et al	Canadá	Enfermería (Pediatria)	0.98	multidimensional
22	Échelle Douleur Inconfort Nouveau-Né revised (EDIN6)	Raffaeli et al	Italia	Medicina Neonatología)	0.9	Unidimensional

**Tabla 2:** Clasificación de las escalas para la valoración del dolor neonatal

ESCALA	DESCRIPCIÓN
Escalas Unidimensionales	
Neonatal Facial Coding system (NFCS)	Esta escala se utiliza para evaluar los movimientos faciales y las expresiones de la incomodidad que presentan los neonatos ante procedimientos dolorosos.
Douleur aigue chez le nouveau-né a terme et prématuré (DAN)	Esta escala evalúa el dolor en los neonatos utilizando parámetros conductuales, específicamente las respuestas conductuales de los neonatos expuestos a procedimientos dolorosos durante la hospitalización.
Face, legs, Activity, Cry, and Consolability (FLACC)	Esta escala se conoce por el acrónimo FLACC, que corresponde a los ítems que evalúa como: expresión de la cara, movimientos de las piernas, la actividad, el llanto y la consolabilidad en un neonato que presenta dolor
Children's and Infants' Postoperative Pain Scale (CHIPPS)	Los ítems que la componen fueron seleccionados a partir de los patrones fisiológicos de un neonato que experimenta dolor después de un posoperatorio. los ítems seleccionados fueron: el llanto, la expresión facial, la postura del torso, la postura de las piernas y la respuesta motora
The Liverpool Infant Distress Scale (LIDS)	El propósito de esta escala fue medir el nivel de dolor posoperatorio de los neonatos, revelando los cambios comportamentales, permitiendo al personal de enfermería realizar intervenciones eficaces para el abordaje del dolor neonatal.
Llanto, actitud, normorrespiración, tono postural y observación facial (LLANTO)	Esta escala se desarrolló a partir de parámetros conductuales observables y está compuesta por 5 ítems que son: el llanto, la actitud, la normo respiración, el tono postural y la observación facial.
Échelle DouleurInconfort Nouveau-Né (EDIN)	Esta escala evalúa el dolor neonatal identificando las siguientes respuestas conductuales: actividad facial, movimientos corporales, calidad del sueño, calidad del contacto con la enfermera, y la consolabilidad.
(A) pitch of the first cry; (B) rhythmicity of the crying bout; (C) constancy of crying intensity (ABC)	Esta escala evalúa la agudeza del primer llanto, el ritmo del estallido, y la constancia de su intensidad. Para probar la sensibilidad y validez concurrente de esta escala, se compararon los resultados con la escala DAN, mostrando resultados similares indicando una buena sensibilidad.
Behavioral Indicators of Infant Pain (BIIP)	Los aspectos que evalúa fueron validados para el dolor en el prematuro, mientras el componente de las expresiones faciales fue validado para medir el dolor posoperatorio del neonato prematuro y a término.
COMFORT B	Originalmente la escala fue diseñada para medir el dolor en los recién nacidos; pero la versión de COMFORT B eliminó los ítems fisiológicos para aumentar la confiabilidad al evaluar el dolor neonatal.
Astrid Lindgren Children's Hospital Pain Scale (ALPS NEO)	Esta escala se desarrolló a partir de una versión existente de la escala (ALPS); para la versión ALPS NEO, se ajustaron los parámetros y se agregó la edad gestacional del neonato y su estado de sedación.
Échelle Douleur Inconfort Nouveau-Né revised (EDIN6)	Esta es una versión revisada y modificada de la escala EDIN, sólo se agregó el ítem de la edad gestacional, mostrando la nueva versión buena confiabilidad al momento de ser usada. Para probar la utilidad clínica de EDIN6, se usó un cuestionario para evaluar la percepción de las enfermeras frente al uso de las escalas EDIN y EDIN6, concluyendo que la escala EDIN6 resulta ser más útil para el ámbito clínico.
Escalas Multidimensionales	
COMFORT	Esta escala utiliza variables fisiológicas (presión arterial media, frecuencia cardiaca y frecuencia respiratoria); y variables comportamentales (estado de alerta, movimientos físicos, tono muscular, tensión facial y el llanto), para valorar el dolor neonatal
The Infant Body Coding System (IBCS)	Para su desarrollo se evaluó a un grupo de neonatos expuestos al procedimiento de punción de talón, comparando las reacciones durante y después del procedimiento.
ESCALA	DESCRIPCIÓN
	Escalas Multidimensionales
C-Crying; R-Requires increased oxygen administration; I-Increased vital signs; E-Expression; S- Sleeplessness. (CRIES)	Se desarrolló basándose en signos conductuales como el llanto y la expresión facial; los desarrolladores de esta escala también consideraron aspectos fisiológicos, para dar precisión a la evaluación del dolor
Premature Infant Pain Profile (PIPP)	Puede utilizarse para evaluar el dolor en bebés prematuros y a término y se compone de los siguientes indicadores: edad gestacional, el estado de la conducta (factores contextuales), la saturación de oxígeno (indicadores fisiológicos) y tres aspectos de la acción facial (indicadores conductuales).
Scale for use in Newborns (SUN)	La intención de su diseño fue proporcionar mayor precisión en la evaluación del dolor que las versiones consultadas. La propuesta planteada por la Scale for use in Newborns es medir tanto parámetros conductuales como parámetros fisiológicos.
Neonatal pain, agitation and sédation Scale (N-PASS) Nepean Neonatal Intensive Care Unit Pain Assessment Tool (NNICUPAT)	Esta escala permite evaluar el dolor y el grado de sedación de los neonatos, teniendo en cuenta la edad gestacional. Esta escala está diseñada para neonatos que se encuentran ventilados midiendo aspectos conductuales y fisiológicos. Para validarla, se utilizó como base otras escalas existentes como la escala CRIES y la escala NFCS.
Multidimensional Assessment of Pain Scale (MAPS)	Consta de 36 elementos de tipo conductual y fisiológico que se agrupan en 5 categorías que son: signos vitales, patrón respiratorio, expresiones faciales, movimientos corporales y estado de excitación.
Pain assessment Scale for preterm infants (PASPI) Premature Infant Pain Profile revised (PIPP-R)	La Escala de evaluación del dolor para lactantes prematuros se aplicó en neonatos de diferentes edades gestacionales para identificar 10 indicadores de dolor. Para validar la escala se comparó con la escala original (PIPP), en este proceso los ítems, fueron conservados, pero las modificaciones solo fueron realizadas en las puntuaciones al momento de abordar la edad gestacional para darle mayor validez.

**Tabla 3:** Escalas desarrolladas por enfermería para valorar el dolor neonatal

Nombre de la escala	Autor/es	País de origen	Especialidad de enfermería
Premature Infant Pain Profile (PIPP)	Stevens et al	Canadá	Pediatría
Face, legs, Activity, Cry, and Consolability (FLACC)	Merkel et al	Estados unidos	Anestesiología
The Liverpool Infant Distress Scale (LIDS)	Horgan et al	Inglaterra	Pediatría
Échelle Douleur Inconfort Nouveau-Né (EDIN)	Debillon, et al	Francia	Pediatría
Multidimensional Assessment of Pain Scale (MAPS)	Ramelet et al	Australia	Materno infantil
Pain Assessment Scale for preterm infants (PASPI)	Liaw et al	Taiwán	Pediatría
Premature Infant Pain Profile revised (PIPP-R)	Stevens et al	Canadá	Pediatría

**Tabla 4:** Estudios de validación identificados

Escala	Autor/res	Muestra de estudio	Idioma de publicación del estudio primario	Adaptación cultural a otro contexto e idioma	Estudios existentes
Neonatal Facial Coding system (NFCS)	Grunau & Craig	140 RN	Inglés	Inglés	Peters et al 2003 (Holanda)
				Portugués	Pereira et al 1999 (Brasil)
COMFORT	Ambuel et al	37 RN	Inglés	Inglés	Van Dijk et al 2000 (Holanda)
Escala	Autor/res	Muestra de estudio	Idioma de publicación del estudio primario	Adaptación cultural a otro contexto e idioma	Estudios existentes
C--Crying; R--Requires increased oxygen administration; I--Increased vital signs; E--Expression; S--Sleeplessness. (CRIES)	Susan W. Krechel Md And Judy Bildner	24 RN a término	Inglés	Inglés	McNair et al 2004 (Canadá)
				Español	Grijalva & Helblin 2015 (Ecuador)
Premature Infant Pain Profile (PIPP)	Stevens et al Stevens et al 2010 (Canadá); Jonsdottir et al 2005 (Islandia)	237 RN	Inglés	Inglés	Ballantyne et al 1999 (Canadá)
Jonsdotti2005 (Islandia)r et al	Stevens et al 2010 (Canadá)			Noruego	Vederhus et al 2006 (Noruega)
Douleur aigue chez le nouveau-né a terme et prématuré (DAN)	Carbajal et al	42 RN	Francés	Inglés	Carbajal et al 1997 (Francia)
Face, legs, Activity, cry, and Consolability (FLACC)	Merkel et al	98 niños de	Inglés	Inglés	Crellin et al 2015 (Australia)
		2 meses a 7 años			Manworren et al 2003 (U.S.A)
Children's and Infants' Postoperative Pain Scale (CHIPPS)	Buettner & Finke	149 RN	Alemán	Inglés	Suraseranivongse et al 2006 (Tailandia)
				Portugués	Alves et al. 2008 (Brasil)
Llanto, actitud, normorrespiración, tono postural y observación facial (LLANTO )	Herrera et al	54 niños	Español	Español	Reinoso-Barbero et al 2010 (España)
		(1 mes-6 años)			Tibaduiza & Ulloa 2015 (Colombia)
Neonatal pain, agitation and sedation Scale (N-PASS)	Hummel et al	72 RN	Inglés	Inglés	Hummel et al 2010 (Estados Unidos)
					Giordano et al 2014 (Austria)
Échelle Douleur Inconfort Nouveau-Né (EDIN)	Debillon et al	76 RN prematuros	Inglés	Inglés	Ancora et al 2009 (Italia)
				Portugués	Barbosa et al 2014 (Brasil)

El 55% de las escalas han sido validadas por medicina, el 37% por el área de enfermería (Tabla 3), el 7% restante por psicología. Del total de escalas identificadas el 55% se clasifican como escalas unidimensionales y el 45% son clasificadas como escalas multidimensionales (Tabla 2).

## Discusión

Esta revisión integrativa proporciona a los profesionales de salud, y en especial al profesional de enfermería bases conceptuales para la implementación de herramientas de evaluación clínica del dolor, según la edad gestacional, la duración del dolor.

Las escalas identificadas para valorar el dolor neonatal miden parámetros fisiológicos, que incluyen la frecuencia cardíaca, frecuencia respiratoria, saturación de oxígeno, presión intracraneal y arterial; entre otras variables, estos parámetros fisiológicos tienen la ventaja de ser mediciones objetivas, sin embargo, hay que tener en cuenta que los cambios de estos parámetros pueden estar relacionados o no a los estímulos dolorosos, situación que permite ampliar un abanico de posibilidades para evaluar el dolor neonatal en condiciones específicas como sedación, post intervención quirúrgica, entre otras situaciones.

Actualmente, hay un número considerable de escalas para evaluar el dolor en los recién nacidos, muchos de los cuales han sido diseñados para la investigación y no para su uso en la práctica clínica^21^, Las escalas relacionadas con comportamiento se adaptan mejor al contexto y la realidad de la práctica clínica diaria, ya que son más fáciles de aplicar^22^ dado que los respectivos indicadores de evaluación son más específicos^23^^-^^25^.

En enfermería se ha desarrollado el 36% de estas escalas, especialmente en las áreas de pediatría y neonatología. Esta revisión integrativa muestra como en los últimos 20 años, la enfermería ha desarrollado 7 escalas para valorar el dolor neonatal todas publicadas en idioma inglés; de éstas, 4 escalas han sido validadas a otros contextos culturales e idiomas. La primera referencia documentada en la revisión de la literatura fue identificada en Canadá en 1996, esta propuesta la desarrollaron las enfermeras pediátricas Stevens, Bonnie; Johnston, Celeste; Petryshen, Patricia; Taddio, Anna^26^; entre la década de los 90 y los años 2000.

La mayor proporción de los estudios primarios de las escalas para valorar el dolor neonatal se trabajó con neonatos a término y pretérmino^17^^,^^18^^,^^24^^,^^26^^-^^29^; sin embargo, algunas escalas valoran el dolor en los lactantes y los pacientes pediátricos^19^^,^^30^^,^^31^, bajo condiciones de sedación^28^^,^^32^^,^^33^ X y durante un posoperatorio^17^^,^^34^^-^^36^, Arias. 2012^37^, señala la variabilidad existente entre las escalas utilizadas para valorar el dolor neonatal, indicando que muchas de las escalas disponibles no especifican el tipo dolor a medir. Asimismo, Olsson et al. 2020^38^, señala que existen escalas con ítems similares, pero cada una de ellas presenta su propia particularidad. Independientemente de la validación con la que cuenten estas escalas, la recomendación para su uso está específicamente orientada según el tipo de dolor y las circunstancias bajo las cuales fueron diseñadas para evaluar a los neonatos con dolor^39^.

El uso de instrumentos para medir y registrar el dolor en el recién nacido según Carvalho y Carvalho^40^, promueve la concientización en el profesional que atiende a esta población, contribuyendo en la mejora de la atención de enfermería.

La Academia Americana de Pediatría, destaca la importancia de evaluar el dolor neonatal, especialmente durante y después de los procedimientos diagnósticos y terapéuticos^41^, con el propósito de controlar la eficacia de las intervenciones y proporcionar un adecuado alivio del dolor. Según su declaración, recomienda 5 escalas a saber: NFCS, PIPP, N-PASS, Indicadores conductuales del Dolor Infantil (BIIP) y Dolor Agudo del Recién Nacido/Douleur Aigue du Nouveau-né (APN/DAN).

Otra escala ampliamente utilizada, no incluida dentro de las 5 sugeridas por la American Academy of Pediatrics, es la escala Face, Legs, Activity, Cry and Consolability (FLACC); debido a que una revisión sistemática muestra que presenta datos limitados y contradictorios para evaluar el dolor ante procedimientos, dada la falta de datos para respaldar el uso de la escala FLACC de manera rutinaria y en todas las poblaciones en las que se aplica actualmente^42^.

Solamente las escalas N-PASS y COMFORT evalúan el dolor y la sedación. Los autores de estas escalas, demostraron que los signos vitales no son representativos para la evaluación del dolor y la sedación. Esta escala utiliza variables comportamentales y fisiológicas para valorar el dolor neonatal; así pues, los ítems que la componen fueron evaluadas por 20 enfermeras experimentadas en el cuidado neonatal, identificando 8 variables de importancia clínica, que son: variables fisiológicas (presión arterial media, frecuencia cardiaca y frecuencia respiratoria); variables comportamentales (estado de alerta, movimientos físicos, tono muscular, tensión facial y el llanto), cada una de ellas tiene calificación máxima de 5 puntos. La validación de esta escala se realizó a través de la concordancia entre evaluadores, presentando un coeficiente interevaluador de 0.90. De igual forma, sus autores realizaron también validación concurrente con enfermeras expertas de unidades cuidados intensivos pediátricos^33^^,^^43^.

Puede existir dificultad por parte del personal de enfermería utilizar las escalas, debido a que no todas evalúan los mismos parámetros; es por ello que, identificar su clasificación permite orientar a un evaluador y escoger el tipo de escala que debe utilizar. Además, la traducción y validación transcultural, así como la aplicación y la evaluación de los efectos son pasos importantes que hay que realizar cuando se aplica una escala determinada en la práctica clínica diaria^44^^,^^45^.

Valorar el dolor en los neonatos permite establecer medidas terapéuticas en su abordaje. En el cuidado de enfermería, para el manejo del dolor se utilizan medidas farmacológicas y no farmacológicas^46^^,^^47^, constituyéndose en elemento fundamental para el cuidado humanizado y la atención integral de los neonatos^8^, especialmente aquellas encaminadas a brindar confort y bienestar del recién nacido^48^. Reconocer el dolor en ellos es indispensable para poder elegir las intervenciones necesarias para su cuidado integral. Así, desde el ámbito clínico se puede evidenciar cómo el personal de enfermería es consciente del dolor neonatal. Sin embargo, el uso de estas escalas, no es frecuente en la práctica de enfermería, porque las instituciones de salud carecen de protocolos que indiquen o recomienden la valoración del dolor neonatal de manera rutinaria.

Finalmente, se pudo identificar que en el contexto colombiano existe un limitado número de estudios que documenten la validación de las escalas para valorar el dolor neonatal^19^.

## Conclusiones

Partiendo del conocimiento de las características de cada escala, no se puede establecer claramente que una u otra sea la más adecuada, ya que la elección por parte del prestador de servicios de salud o del investigador dependerá de la edad gestacional, del tipo de estímulo doloroso y del contexto en el que se encuentra el recién nacido por tanto finalizada la revisión se identifica que no existe una escala de referencia para la evaluación del dolor en los recién nacidos. El profesional de la salud y en especial el personal de enfermería, debe utilizar escalas validadas, fiables, seguras y prácticas en los neonatos, ya sean escalas unidimensionales o multidimensionales, teniendo en cuenta la variabilidad encontrada en la literatura.

Se destaca que la evaluación del dolor en el período neonatal debe ser multidisciplinaria; dada la subjetividad del fenómeno evaluado y de las escalas disponibles, cuando más profesionales de diferentes áreas de la salud evalúan al mismo recién nacido utilizando diferentes escalas, tal vez se pueda aumentar la objetividad de esta evaluación.

La adopción de escalas de valoración del dolor desde la experiencia clínica debe contribuir a la actualización y utilización de rutinas y protocolos que contribuyan a la evaluación y tratamiento del dolor en el recién nacido. Así mismo, la formación y cualificación de los profesionales que trabajan en estas unidades, deben garantizar la aplicación práctica de los conocimientos relacionados con la prevención, la evaluación y el manejo del dolor, con el fin de estandarizar la actuación de los profesionales del servicio y permitir un tratamiento adecuado de los neonatos.
